# Association between Medical Comorbidities and White Matter Hyperintensity Volume in Diverse Populations: A HABS‐HD Study

**DOI:** 10.1002/alz.093425

**Published:** 2025-01-09

**Authors:** Antoine R Trammell, James J. Lah, Monica W Parker, Felicia Goldstein, Chadwick M Hales, Crystal P Davis, Cornelya D Dorbin, Sabria Saleh, Elizabeth Murphy‐Tchivzhel, Alecia Michella Rose, Amber Osborne, Adiva Ahmad, Jahmila Al‐Amin, Sid E. O'Bryant

**Affiliations:** ^1^ Emory University Goizueta Alzheimer's Disease Research Center, Atlanta, GA USA; ^2^ Emory University, Atlanta, GA USA; ^3^ Emory University School of Medicine, Atlanta, GA USA; ^4^ University of North Texas Health Science Center, Fort Worth, TX USA

## Abstract

**Background:**

Older African American (AA) and Hispanic American (HA) adults have a higher risk for Alzheimer’s disease and related dementias (ADRD) compared to non‐Hispanic white (NHW) adults. AA and HA persons may also develop disease at younger ages with more rapid progression. Vascular disease, including cerebral small vessels, manifest by white matter hyperintensity (WMH) lesions, is more prevalent in AA and HA. Evidence suggests that vascular disease and WMH lesions may influence ADRD risk. This study explores WMH volume and vascular health conditions in a diverse research sample.

**Method:**

We obtained demographic, clinical history, laboratory, and brain magnetic resonance imaging data collected from Health and Aging Brain Study: Health Disparities baseline visits. Using ANOVA and chi‐square, we compared conditions with ADRD risk (depression, hypertension, diabetes, hyperlipidemia, stroke), blood pressure, body mass index (BMI), and labs by self‐reported race (NHW, AA, HA). We performed linear regression adjusted for age, education, and sex to evaluate associations between WMH volume and clinical conditions.

**Result:**

Data from 3,035 participants was analyzed (mean age: 65.2; female: 62.3%; impaired: 20.1%). AA were younger (NHW: 68.6, AA: 62.9, HA: 62.3. p<.0001) and had higher BMI (NHW: 29.1, AA: 32.5, HA: 30.9, p<.0001). Among conditions, HA had more hypertension (NHW: 18.2%, AA: 15.3%, HA: 19.9%, p<.0001), diabetes (NHW: 4.68%, AA: 4.71%, HA:12.4%, p<.0001), higher A1c (NHW: 5.57, AA: 6.02, HA: 6.43, p<.0001), and higher triglycerides (NHW:120, AA: 99.2, HA: 152, p<.0001). NHW had more depression (NHW:10.4%, AA: 4.88%, HA: 8.76%, p=0.006). AA had the highest WMH volume (NHW: 4.16, AA: 18.4, HA: 3.18, p<.0001). Regression showed higher WMH volume was associated with BMI in AA (β=.33, p=.007) and HA (β=1.84, p=.001). In AA, higher WMH volume was associated with diabetes (β=23.7, p=.02) and HbA1c (β=8.32, p=.02). In NHW, higher WMH volume was associated with stroke (β=4.76, p<.0001) and depression (β=1.18, p=.007).

**Conclusion:**

Study findings show associations between WMH burden and medical conditions, labs, and physical measures. Results also show that cerebral small vessel disease and cognitive impairment risk factors may vary by race. Study findings highlight the benefit of diverse research samples in gaining further insights into ADRD risk, pathogenesis, and disease progression.

